# Radiation‐associated sarcoma after breast cancer in a nationwide population: Increasing risk of angiosarcoma

**DOI:** 10.1002/cam4.1698

**Published:** 2018-07-25

**Authors:** Samuli H. Salminen, Mika M. Sampo, Tom O. Böhling, Laura Tuomikoski, Maija Tarkkanen, Carl P. Blomqvist

**Affiliations:** ^1^ Comprehensive Cancer Center Helsinki University Hospital (HUH) and University of Helsinki Helsinki Finland; ^2^ Department of Pathology University of Helsinki and HUSLAB Helsinki University Hospital Helsinki Finland; ^3^ Department of Oncology Örebro University Hospital Örebro Sweden

**Keywords:** breast neoplasms, hemangiosarcoma, radiotherapy, registries, sarcoma

## Abstract

Radiation‐associated sarcoma (RAS) is a rare complication of radiation therapy (RT) to breast cancer (BC). This study explored RAS after RT to BC in a nationwide population‐based material. The Finnish Cancer Registry was queried for patients with BC treated during 1953‐2014 who were later diagnosed with a secondary sarcoma in 1953‐2014. Registry data, patient files, and sarcoma specimens were  analyzed to confirm diagnosis and location of RAS at or close to the RT target volume. A total of 132 512 patients were diagnosed with invasive BC during the study period. A subsequent sarcoma was diagnosed in 355 patients. After exclusion, 96 RAS were identified. Angiosarcoma (AS) was the most prevalent histology in 50 (52%) of 96 patients. However, the first radiation‐associated AS was diagnosed in a patient treated for BC with breast‐conserving surgery in 1984, and thereafter, the proportion of AS continuously increased. The 5‐year sarcoma‐specific survival was 75.1% for RAS treated with a curative intent. The distribution of histologic subtypes of RAS has changed during the 60 years of this registry study. The first radiation‐associated AS was diagnosed in 1989, and presently, AS is the most common histologic subtype of RAS. It is possible that changes in BC treatment strategies are influencing the characteristics of RAS.

## 1 INTRODUCTION

Breast cancer (BC) comprised 23% of global cancer incidence and 14% of global cancer mortality among women in 2008 showing an increase in both incidence and mortality worldwide by the year 2012.^1^ Most BCs are diagnosed at an early stage for which the gold standard of treatment is breast‐conserving surgery combined with radiation therapy (RT).^2^

The side effects of RT are typically transient skin reactions, but in rare cases, RT is also associated with the development of sarcoma (radiation‐associated sarcoma [RAS]). The 15‐year cumulative incidence of RAS was 0.28% among patients with BC treated during 1954‐1983.^3^ A significant relationship between radiation dose and the risk of subsequent sarcoma has been reported, with a relative risk of 30.6 for doses higher than 44 Gy compared to 14 Gy or lower.^3^ Cahan et al^4^ defined RAS arising in bone as sarcoma diagnosed in the RT field after a relatively long asymptomatic period before diagnosis of RAS with histologic proof of sarcoma. These criteria were later modified to include tissues adjacent to the path of the radiation beam at risk of the development of RAS and a latency period of at least 3‐4 years.^5^ The nomenclature in the literature is not always conclusive. The term RAS is sometimes used also in cases where RAS location has not been analyzed in relation to RT target volume.^6^ For this study, we define RAS as a sarcoma arising at or close to the RT target volume, and postirradiation sarcoma (PIS) as sarcoma arising in any location in an individual with the previous history of RT. Therefore, the incidence of PIS is higher than that of RAS.

The distribution of histologic subtypes among RAS differs from sporadic sarcomas. Angiosarcoma (AS) has been the most common subtype in recent studies,^7^, ^8^, ^9^, ^10^, ^11^ while osteosarcoma and undifferentiated pleomorphic sarcoma (UPS) have been the most common types in older series.^12^

The main aim in this study was to assess time trends in clinical characteristics and histologic subtypes of RAS after RT to invasive BC in a nationwide Finnish population between 1953 and 2014 exploiting registry data, detailed data from patient files, and histologic review.

## 2 PATIENTS AND METHODS

### 2.1 Finnish Cancer Registry

The Finnish Cancer Registry (FCR) was established in 1952, and it covers the whole population of Finland with approximately 5.6 million inhabitants presently. In 1961, the National Board of Health issued a by‐law making reporting of cancers to FCR compulsory. All physicians, hospitals, and other institutions are obliged to send a notification of all diagnosed cancer cases. Pathology, cytology, and hematology laboratories send a separate laboratory notification, mostly electronically nowadays. In addition, FCR contacts Statistics Finland annually to receive all death certificates where cancer is mentioned. FCR database includes close to complete national cancer data for solid malignant tumors diagnosed since 1953, which can be used for statistical and research purposes.^13^ In 2008, FCR changed to the ICD‐O‐3 (International Classification of Diseases for Oncology) coding system^14^ and converted earlier codes (based on ICD7) to match the ICD‐O‐3. In Finland, all inhabitants have a unique personal ID code enabling reliable patient identification and follow‐up. The FCR contains data on patient identity, primary tumor site and date of diagnosis, diagnostic methods, tumor morphology, and, if notified, also stage of the disease and primary treatment. Possible date and cause of death or emigration are checked and included on annual basis.

### 2.2 Patient selection

**Figure 1:**
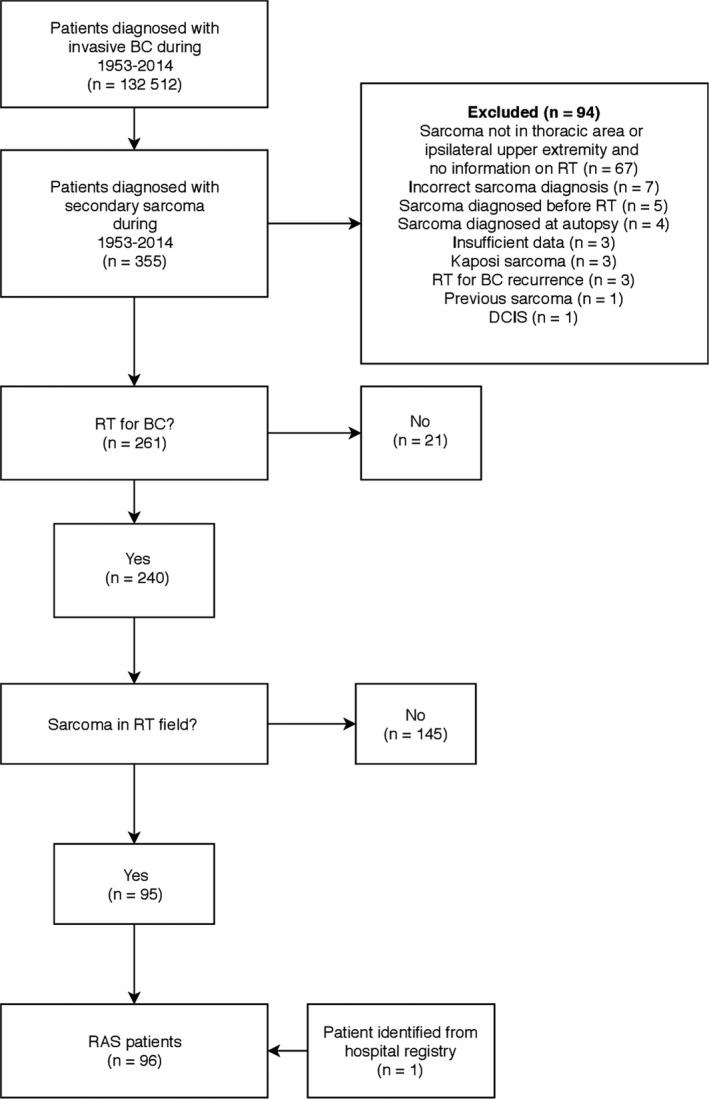
Flowchart of the study population; BC, breast cancer; DCIS, ductal carcinoma in situ; RAS, radiation‐associated sarcoma; RT, radiotherapy

Public healthcare system is mainly responsible for cancer diagnostics and treatment in Finland. Mammography‐based screening for BC started in 1987 with high compliance.^15^ After approval from the Joint Ethics Committee of Helsinki University Hospital and from the Ministry of Health and Social Affairs, we searched the files of the FCR for patients diagnosed with an invasive breast carcinoma (ICD‐O‐3^14^ morphology codes 801.0, 801.3, 802.0, 802.1‐802.2, 803.2, 804.1, 807.0, 807.4, 808.2, 812.3, 814.0‐814.1, 820.0, 820.1, 821.1, 824.0, 824.6, 824.9, 825.5, 831.0, 832.3, 840.1, 844.0, 848.0, 850.0‐850.4, 851.0, 852.0, 852.2, 852.3, 852.4, 853.0, 854.0‐854.1, 854.3, 855.0, 856.0, 857.2, 857.5 with behavior 3 and topography codes C50.0‐C50.9) between 1953 and 2014 (n = 132 512; Figure 1). The FCR database was then queried for secondary sarcomas occurring in these patients (ICD‐O‐3 morphology codes 880.0‐893.6, 896.3‐896.4, 898.0‐898.1, 899.0‐899.1, 902.0, 904.0‐904.4, 912.0‐914.0, 917.0‐924.3, 926.0‐934.2, 953.5 with behavior 3; malignant, primary site) during 1953‐2014 (n = 355). No minimum latency period was set. Registry data, patient records and pathology reports were used to assess eligibility for the study. Treatment of BC and precise location of the sarcoma were analyzed from patient records to ensure that RT was used in BC treatment and that RAS was at or close to the RT target volume. In cases where sarcoma was not directly in RT target volume, treatment plans were reviewed to confirm that the sarcoma was at least partially located inside the irradiated volume. The study period overlaps with a study from our institution including seven RAS patients after BC between 1953 and 1988.^12^ Of these seven patients, we excluded one patient because RT was administered to BC local recurrence instead of primary BC (Figure 1).

### 2.3 BC features

Detailed patient, tumor, and treatment characteristic data of BC were obtained from patient records and pathology reports. Histology (ductal, lobular, or other), grade (1‐3), estrogen‐ and progesterone receptor status (positive >10% by immunohistochemistry), HER‐2 receptor overexpression (positive by in situ hybridization), and nodal involvement (present or absent) were recorded. Surgery (resection or mastectomy), chemotherapy (yes or no), endocrine therapy (yes or no), axillary RT (yes or no), tumor bed RT boost (yes or no), and RT maximum dose and dose per fraction were recorded.

### 2.4 Histologic reevaluation of RAS

All RAS samples were subjected to a histologic reevaluation by an experienced sarcoma pathologist (T.B.). We were unable to obtain the histologic specimens in eight RAS cases. Of these, five were AS cases diagnosed during 1989‐2014 and three were RAS of other histologic subtypes diagnosed during 1994‐2012. These patients were nevertheless included in the study based on the original pathology report, which on review was considered diagnostic by our reviewer (T.B.). Lesions were graded according to the French grading system.^16^ One patient was excluded because diagnosis was changed from sarcoma to premalignant vascular lesion in the reevaluation.

### 2.5 Follow‐up

We defined the latency period as the interval from the first day of RT to BC to the histologic diagnosis of the RAS. Survival data were calculated from the histologic diagnosis of RAS to the last follow‐up. We recorded both local recurrences and systemic recurrences from RAS and BC together with data on possible death and death cause.

### 2.6 Statistical analyses

The number of expected cases was calculated based on national incidence rates and person‐years at risk, stratified by age groups (5‐year intervals), year of sarcoma diagnosis (5‐year interval), and sex. The standardized incidence ratio (SIR) for secondary sarcoma after diagnosis of breast cancer was calculated as the ratio of the observed number of cases to the expected number of cases. Sarcoma‐specific survival (SSS) was defined as the time from histologic diagnosis of RAS to death from RAS or treatment complication with censoring at the last follow‐up date. SSS rates were calculated with the Kaplan‐Meier method. IBM^®^ SPSS^®^ Statistics for Windows, Version 24.0. (Armonk, NY: IBM Corp.) and the R Project for Statistical Computing were used for all analyses.

## 3 RESULTS

A total of 132 512 patients were diagnosed with an invasive BC during 1953 and 2014 (Figure 1), and this yielded a total of 1 257 946 patient‐years at risk. Of these patients, 355 patients were diagnosed with a subsequent sarcoma during the study period vs 187.72 expected (SIR 1.89 [95% confidence intervals 1.7‐2.09]). Seventy‐four ASs were diagnosed vs 6.84 expected (SIR 10.81 [95% CI 8.53‐13.47]), and 281 other secondary sarcomas were diagnosed vs 180.88 expected (SIR 1.55 [95% CI 1.38‐1.74]). A table describing the observed and expected numbers of sarcomas during the study period is listed in Appendix 1. Only four secondary AS were diagnosed in patients treated for BC during 1950‐1979 (SIR ranging from 0 to 13.2). In contrast, 69 secondary AS were found in patients treated for BC during 1980‐2009 (SIR ranging from 4.92 to 19.81).

**Figure 2:**
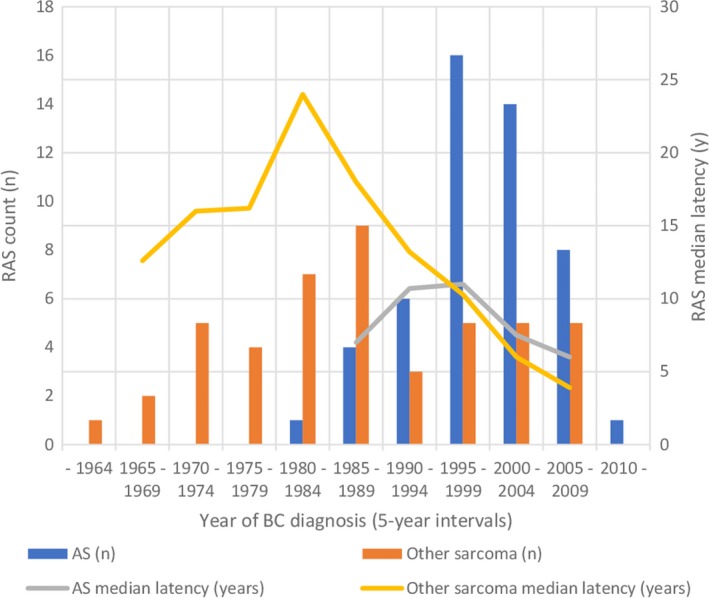
Trends in RAS incidence and median latency; AS, angiosarcoma; BC, breast cancer; RAS, radiation‐associated angiosarcoma

After exclusion of cases not fulfilling the criteria for RAS, 96 RAS patients remained and comprised the study population (Figure 1). The mean age (SD) at BC and RAS diagnosis was 56 (11) and 68 (10) years, respectively. Fifty‐two (54%) patients with BC were operated with breast‐conserving surgery (Table 1). The year 1985 was a turning point in the surgical technique as only 10% of patients were operated with breast‐conserving surgery before 1985 and 66% after 1985. The total RT dose to BC varied from 28 to 60 Gy and fraction size from 1.8 to 5 Gy. Most patients received a total dose of 50 Gy (n = 52) in 2‐Gy fractions (n = 54). AS was the most common histologic subtype among RAS patients, accounting for 52% (50 of 96 patients) of RAS (Table 1). However, no AS was diagnosed during the first three decades of study (Figure 2). Instead, the first AS in our series was diagnosed in a patient receiving RT to BC in 1984, and the proportion of AS increased thereafter (Figure 2). All but one RAS were of intermediate or high grade (Table 2). Resected breast was the most common location of RAS (46 of 96 cases). AS was the most common histologic subtype (37 of 46 patients) in the resected breast.

**Table 1:** Breast cancer and treatment characteristics of the 96 breast cancer patients by radiation‐associated sarcoma

Characteristic	Total no. of patients, n (%)	Angiosarcoma, n (%)	Other sarcoma, n (%)	*P* a
	96	50	46	
Surgery type				
Mastectomy	44 (46)	11 (22)	33 (72)	<0.05
Resection	52 (54)	39 (78)	13 (28)	
Primary BC tumor size				
<20 mm	53 (55)	34 (68)	19 (41)	0.127
≥20 mm	26 (27)	12 (24)	14 (30)	
Missing	17 (18)	4 (8)	13 (28)	
Histology				
Ductal	61 (64)	35 (70)	26 (57)	<0.05
Lobular	14 (15)	10 (20)	4 (9)	
Other	15 (16)	4 (8)	11 (24)	
Missing	6 (6)	1 (2)	5 (11)	
Grade				
1	19 (20)	16 (32)	3 (7)	<0.05
2	41 (43)	26 (52)	15 (33)	
3	9 (9)	2 (4)	7 (15)	
Missing	27 (28)	6 (12)	21 (46)	
Node status				
Negative	53 (55)	34 (68)	19 (41)	<0.05
Positive	42 (44)	16 (32)	26 (57)	
Missing	1 (1)	0	1 (2)	
Estrogen receptor status				
Negative	8 (8)	3 (6)	5 (11)	0.439
Positive	57 (59)	38 (76)	19 (41)	
Missing	31 (32)	9 (18)	22 (48)	
Progesterone receptor status				
Negative	17 (18)	12 (24)	5 (11)	0.316
Positive	46 (48)	29 (58)	17 (37)	
Missing	33 (34)	9 (18)	24 (52)	
HER2 overexpression				
Negative	31 (32)	20 (40)	11 (24)	1.00
Positive	2 (2)	1 (2)	1 (2)	
Missing	63 (66)	29 (58)	34 (74)	
Adjuvant endocrine therapy				
No	63 (66)	32 (64)	31 (67)	0.840
Yes	33 (34)	18 (36)	15 (33)	
Adjuvant chemotherapy				
No	76 (79)	48 (96)	28 (61)	<0.05
Yes	20 (21)	2 (4)	18 (39)	
Axillary lymph node RT				
No	49 (51)	37 (74)	12 (26)	<0.05
Yes	46 (48)	13 (26)	33 (72)	
Missing	1 (1)	0	1 (2)	
Radiation boost at tumor bed				
No	79 (82)	37 (74)	42 (91)	<0.05
Yes	17 (18)	13 (26)	4 (9)	

HER2, human epidermal receptor 2; RT, radiation therapy.

a Either chi‐squared or Fisher's exact test.

**Table 2:** Radiation‐associated sarcoma and treatment characteristics of the 96 patients by radiation‐associated sarcoma

Characteristic	Total no. of patients	Angiosarcoma, n (%)	Other sarcoma, n (%)
	96	50	46
Site			
Breast	46 (48)	37	9
Upper trunk	16 (17)	7	9
Ablation scar	11 (11)	6	5
Shoulder	6 (6)	0	6
Sternum	5 (5)	0	5
Axilla	4 (4)	0	4
Lung	4 (4)	0	4
Scapula	3 (3)	0	3
Upper arm	1 (1)	0	1
Site			
Soft tissue	88 (92)	50	38
Bone	8 (8)	0	8
Metastases at presentation			
Yes	9 (9)	0	9
No	87 (91)	50	37
Histology			
Angiosarcoma	50 (52)		
UPS	27 (28)		
Osteosarcoma	5 (5)		
Fibrosarcoma	3 (3)		
Extraskeletal osteosarcoma	3 (3)		
Chondrosarcoma	2 (2)		
Leiomyosarcoma	2 (2)		
Myxofibrosarcoma	2 (2)		
Extraskeletal chondrosarcoma	1 (1)		
Neurofibrosarcoma	1 (1)		
Grade			
1	1 (1)	0	1 (2)
2	22 (23)	8 (16)	14 (30)
3	70 (73)	39 (78)	31 (67)
Missing	3 (3)	3 (6)	0
Operated with curative intent			
Yes	82 (85)	47 (94)	35 (76)
No	14 (15)	3 (6)	11 (24)
Adjuvant RT^a^			
Yes	9 (11)	1 (2)	8 (23)
No	73 (89)	46 (98)	27 (77)
Adjuvant CT^a^			
Yes	9 (11)	5 (10)	4 (11)
No	73 (89)	42 (89)	31 (89)

CT, chemotherapy; RAS, radiation‐associated sarcoma; RT, radiation therapy; UPS, undifferentiated pleomorphic sarcoma.

a Of the 82 patients treated with curative intention.

### 3.1 Latency

The median latency of RAS varied depending on the time of BC diagnosis (Figure 2). The median latency was 11.0 years (range 0.6‐29.9) for all RAS, 7.7 years (range 0.6‐24.5) for AS, and 13.8 years (range 2.3‐29.9) for other sarcomas, respectively. For BC patients treated before 1984, the median latency of RAS was 16.4 years (range 11.0‐29.9). For BC patients treated in or after 1984, the median latency was 8.2 years (range 0.6‐27.9) for all RAS, 7.7 years (range 0.6‐24.5) for AS, and 10.6 years (range 2.3‐27.9) for other RAS, respectively.

### 3.2 Treatment and survival

Eighty‐two (85%) RAS patients were operated with curative intent. Nine RAS patients received adjuvant RT, and nine patients received adjuvant CT for RAS. Six BC patients developed systemic BC during follow‐up. Five of these six patients were operated for RAS with curative intent. Median follow‐up for survivors from the diagnosis of RAS was 4.4 years (range 0.1‐32.6 years). At last follow‐up, 46 (48%) of 96 RAS patients were alive with no evidence of BC or RAS. Forty patients died of RAS, four patients died of BC with no evidence of RAS, and four patients died of non‐cancer‐related causes. Two patients were alive with active BC with no evidence of RAS. Five‐year SSS was 64.8% for all patients and 75.1% for patients treated with curative intent. For patients treated with curative intent, the 5‐year SSS was 75.9% for AS and 76.1% for other RAS.

At RAS diagnosis, nine patients presented with metastatic disease. All these patients died of RAS with a median SSS of 0.43 years (range 0.1‐2.8). Twenty‐two patients receiving primary treatment for local RAS with curative intent later developed systemic disease. Twenty patients died of systemic RAS, whereas two patients are alive with no evidence of RAS at 3.1 and 5.1 years after developing the systemic disease. The median SSS calculated from the diagnosis of metastatic disease was 0.6 years (range 0.02‐5.1) for AS and 0.7 years (range 0‐5.1) for other RAS.

## 4 DISCUSSION

The most important finding in this nationwide survey of RAS among 132 512 BC patients treated during 1953‐2014 was the increasing incidence of AS during the last three decades after treatment of BC. During the last three decades of study period, the incidence ratio of AS increased compared to the incidence ratio of other secondary sarcomas. This observation seemed to be due to the striking increment of AS among RAS of the breast. The first radiation‐associated AS was diagnosed in a patient treated for BC in 1984. Thereafter, the incidence of AS steadily increased and in fact AS was the most common (52%) RAS histologic subtype in the current series. In our previous nationwide study on patients with RAS diagnosed during 1953‐1987 after RT for any malignancy including BC, no case of AS was found.^12^

It is crucial to distinguish between true postirradiation sarcomas, that is sarcomas arising at or close to the RT target volume and other secondary sarcomas, as not all previous studies have analyzed the location of the sarcoma in relation to the RT target volume. This is of special importance in BC, as AS related to edema of the ipsilateral arm after surgery and radiation of the axilla has long been recognized as a clinical entity, that is, the Stewart‐Treves syndrome.^17^ The largest study of sarcomas arising after treatment of BC hitherto, the SEER study, unfortunately does not distinguish between sarcomas arising in the RT target volume or elsewhere in the ipsilateral thoracic region or ipsilateral arm.^6^ Only a few cases of true RAS AS after RT for BC have been reported in the medical literature in the 70s and early 80s,^18^ whereas most postirradiation ASs developing before 1980s arose in lymphedematous arms.^19^, ^20^, ^21^, ^22^ The only two AS among 11 sarcomas judged to be induced by irradiation in 7620 patients treated for BC at Institut Gustave Roussy in France during 1954‐1983 were located outside the RT target volume in the upper extremity.^19^ Ferguson et al^20^ reported four non‐AS sarcomas at the treated chest wall and two AS in the swollen ipsilateral arm after mastectomy and irradiation among 211 patients treated during 1927‐1970, and Davidson et al^21^ reported two lymphangiosarcomas situated in irradiated axilla after treatment for BC among the 20 patients diagnosed with RAS at the Royal Marsden Hospital 1954‐1985 after RT to any indication. In a Swedish population‐based study on patients treated for BC during 1958‐1992, only two of 32 AS described as being located “close to breast” were in fact located in the conserved breast whereas 30 were located in the edematous ipsilateral arm.^22^ Thus in these older series of patients with BC treated between 1927 and 1992, only four of 38 postirradiation AS may be classified as RAS according to the definition of Cahan, the majority of AS being due to lymphedema of the ipsilateral arm.

In contrast to older series, as in the present series an increasing number of AS in RT target volume have been reported in patients treated during and after the 1980s. Many patient series with confirmed location of RAS in the RT target volume, however, include only patients with AS and do not report RAS of all histologic subtypes. A few mixed series have, however, been published, and these are summarized in Table 3.^7^, ^8^, ^9^, ^10^, ^11^, ^12^, ^23^, ^24^, ^25^, ^26^, ^27^, ^28^, ^29^, ^30^, ^31^, ^32^, ^33^, ^34^ The proportion of patients treated for BC varies in these series from 8% to 100%. Most AS (66/73) develop in patients treated for BC.^8^, ^9^, ^10^, ^11^, ^23^, ^24^, ^26^, ^27^, ^29^, ^32^ Among patient series with RAS after primary tumor of any location extending in the 2000s, 21% (222/1040) of all cases were AS^7^, ^8^, ^9^, ^10^, ^11^, ^27^, ^28^, ^29^, ^30^, ^31^, ^32^, ^33^, ^34^ compared to 5% (9/176) in series of RAS diagnosed within the 20th century.^12^, ^23^, ^24^, ^25^ Unfortunately, the year of RT is given in only a few series. The five series, where the time of RT was stated, reveal a similar trend as the present study; the proportion of AS in two series with patients irradiated from 1953 to 1988 was 2%,^12^, ^24^ while one series of patients irradiated from 1981 to 1997 reported an AS proportion of 48%.^9^ The two series of patients irradiated from 1961 to 1996 reported an intermediate frequency of AS of 23%.^25^, ^27^

**Table 3:** Patient series reporting all histologic subtypes of in‐target radiation‐associated sarcomas

	RT given	Bone/soft tissue	BC%	RAS diagnosed	Latency required (y)	AS/all		AS in BC patients	
						n	%	n	%
Wiklund et al^12^	1953‐1988	B/S	21	1953‐1988	1	0/33	0	0/7	0
Laskin et al^23^	NA	S	23	1954‐1986	2	1/53	2	0/12	0
Kuten et al^24^	1953‐1978	S	100	1968‐	NA	1/7	14	1/7	14
Pierce et al^25^	1968‐1985	S	100	NA	NA	0/3	0	0/3	0
Lagrange et al^26^	NA	B/S	42	1975‐1995	3	7/80	9	6/34	18
Thijssens et al^27^	1961‐1996	B/S	52	1978‐2003	3	7/27^a^	26	7/14	50
Kalra et al^7^	NA	B	19	1978‐2005	NA	0/42	0	0/8	0
Erel et al^8^	NA	B/S	100	1978‐2009	NA	5/25	20	5/25	20
Bjerkehagen et al^28^	NA	B/S	20	1980‐2008	2	12/106	11	NA	NA
Gladdy et al^29^	NA	S	34	1982‐2007	0.5	27/130	21	22/44	50
Kirova et al^9^	1981‐1997	B/S	100	NA	3	13/27	48	13/27	48
Mavrogenis et al^10^	NA	B/S	8	1985‐2011	3	1/52	2	1/4	25
De Smet et al^30^	NA	B/S	50	1987‐2007	1	17/46	37	NA	NA
Riad et al^31^	NA	S	34	1989‐2009	3	8/44	18	NA	NA
Neuhaus et al^32^	NA	S	51	1990‐2005	3	9/67	13	9/34	27
Penel et al^33^	NA	S	45	1997‐2005	3	4/22	18	NA	NA
Kim et al^11^	NA	B/S	27	2000‐2014	0.5	2/33	6	2/9	22
Zhang et al^34^	NA	B/S	51	2000‐2014	0.5	117/419	28	NA	NA

AS, angiosarcoma; B, bone; BC breast cancer; NA, not available; RAS, radiation‐associated sarcoma; RT, radiation therapy; S, soft tissue.

a First AS diagnosed in 1995.

In a review from the Southern Swedish healthcare region on patients treated with RT for BC, the conclusion was that “the clinical presentation of AS has changed, parallel with altered treatment principles for BC”.^35^ This was based on a finding that of the 31 patients developing AS, 14 females treated during 1949‐1988 developed AS in edematous arms (Stewart‐Treves syndrome) after median latency of 11 years, whereas 17 patients treated during 1980‐2005 developed AS in the irradiated field on the thoracic wall after median of 7.3 years.^35^ A similar result was apparent in a study from the Institut Gustave Roussy, France, by Rubino et al^3^ where 7711 patients treated for BC during 1954‐1983 were analyzed for secondary sarcomas. Three patients treated with mastectomy and external RT during 1970‐1976 developed AS in the upper arm, whereas one patient with BC treated with tumorectomy and RT in 1983 developed an in‐field AS.^3^ Our results are confirmatory with the first in‐field AS occurring in a patient receiving RT to BC during the 1980s, and a steady increase in incidence thereafter.

One key strength of the current study was the truly nationwide material based on the reliable Finnish Cancer Registry with nearly 100% completeness in solid tumors^13^ Furthermore, all patient records were assessed for detailed information. Although our study has many strengths, some weaknesses need to be discussed. Firstly, a small proportion (8%) of RAS samples were not available for reevaluation. Histologic reevaluation of RAS samples yielded exclusion of only one patient with atypical vascular lesion possibly a precursor of AS. Therefore, the impact of missing histologic reevaluation on our results is probably small. Sarcoma, especially AS, has a specific morphology compared to recurrent carcinoma. Thus, we felt comfortable including also unavailable specimen after assessment of the initial pathology reports by an experienced sarcoma pathologist. Another limitation concerning especially the study of time trends is inherent to the characteristics of RAS itself. Mery et al^6^ reported the risk of developing soft tissue sarcoma after RT to BC peaking at 10 years and remaining elevated up to 20 years after RT. The long latency time affects the distribution of latency times in the present series. Latency appears to be misleadingly short for cases at the beginning and end of the follow‐up period due to left and right truncation of the distribution of latency times. This is partially reflected in the variation of RAS median latency shown in Figure 2. Statistical methods utilizing left or right censoring are not applicable in the present study, as the cancer registry does not include reliable data on exposure of RT among BC cases.

The reason for the increase in AS incidence is unclear, but one possible factor is RT administered after breast resection rather than mastectomy. This cannot, however, be the only explanation because AS developed after breast ablation in 11 (11/50 AS) patients. Increased use of medical adjuvant therapy cannot be the reason either, as only 18 AS patients received adjuvant endocrine therapy and two AS patients received cytotoxic treatment. Estimation of the risk of RAS in irradiated BC patients as a function of time is beyond the scope of the current study due to the limitations described above. We can, however, speculate that as the number of patients exposed to RT as part of the treatment for BC increases^6^ and survival for BC improves,^36^ RAS should show an increase in incidence. The current study offers no evidence that the incidence of RAS of other histologic subtypes than AS is increasing.

Our material provides a unique view of RAS after BC in Finland. Patients were identified from a national, comprehensive cancer registry. Our study covers all RAS diagnosed between 1953 and 2014 after BC, providing an extensive time span. BC incidence has increased significantly in Finland (39/100 000 in 1967 and 161/100 000 in 2012), and the 5‐year survival of BC patients improved (53% during 1965‐1969 and 88% during 2010‐2014).^36^ These factors are likely to influence the incidence of RAS in combination with the evolution of RT techniques during past decades. We found that the total number of RAS increased during the last 30 years, which at least partly may relate to a higher number of patients exposed. The most striking finding, however, was the emerging of and continuous increase of AS after BC treated in the 1980s or later, while no increase in the incidence of other histologic types was seen. Further research is required to determine the cause of this change in the histologic distribution of RAS.

## CONFLICT OF INTEREST

The authors made no disclosures.

## Appendix 1 Observed vs expected secondary sarcomas during the study period

### 1.1

**Table:**

BC diagnosis	Angiosarcoma				Other sarcoma			
	Observed	Expected	Person‐years	SIR (95% CI)	Observed	Expected	Person‐years	SIR (95% CI)
1950‐1954	0	0.02	11688.5	0 (0‐Inf)	1	1.30	11688.5	0.77 (0.11‐5.44)
1955‐1959	1	0.08	31951.8	13.12 (1.85‐93.14)	1	3.54	31951.8	0.28 (0.04‐2)
1960‐1964	0	0.12	40024.2	0 (0‐Inf)	8	4.72	40024.2	1.69 (0.85‐3.39)
1965‐1969	1	0.19	51755.4	5.15 (0.73‐36.57)	11	6.50	51755.4	1.69 (0.94‐3.06)
1970‐1974	0	0.27	69831.6	0 (0‐Inf)	18	9.17	69831.6	1.96 (1.24‐3.11)
1975‐1979	2	0.34	87673.0	5.81 (1.45‐23.24)	28	11.99	87673.0	2.34 (1.61‐3.38)
1980‐1984	4	0.51	110413.6	7.82 (2.94‐20.85)	22	15.85	110413.6	1.39 (0.91‐2.11)
1985‐1989	4	0.81	146975.6	4.92 (1.85‐13.11)	39	21.88	146975.6	1.78 (1.3‐2.44)
1990‐1994	11	0.99	168322.8	11.12 (6.16‐20.07)	30	25.09	168322.8	1.2 (0.84‐1.71)
1995‐1999	22	1.11	184461.7	19.81 (13.04‐30.08)	42	27.41	184461.7	1.53 (1.13‐2.07)
2000‐2004	16	1.11	173883.8	14.4 (8.82‐23.51)	42	25.99	173883.8	1.62 (1.19‐2.19)
2005‐2009	12	0.90	128567.1	13.3 (7.55‐23.42)	29	19.21	128567.1	1.51 (1.05‐2.17)
2010‐2015	1	0.39	52396.9	2.59 (0.36‐18.37)	10	8.22	52396.9	1.22 (0.65‐2.26)

BC, breast cancer; CI, confidence interval; Inf, infinity; SIR, standardized incidence ratio.
